# Black Liquor and Wood Char-Derived Nitrogen-Doped Carbon Materials for Supercapacitors

**DOI:** 10.3390/ma16072551

**Published:** 2023-03-23

**Authors:** Loreta Tamasauskaite-Tamasiunaite, Jolita Jablonskienė, Dijana Šimkūnaitė, Aleksandrs Volperts, Ance Plavniece, Galina Dobele, Aivars Zhurinsh, Vitalija Jasulaitiene, Gediminas Niaura, Audrius Drabavicius, Mari Juel, Luis Colmenares-Rausseo, Ivar Kruusenberg, Kätlin Kaare, Eugenijus Norkus

**Affiliations:** 1 Center for Physical Sciences and Technology (FTMC), LT-10257 Vilnius, Lithuania; 2 Latvian State Institute of Wood Chemistry, LV-1006 Riga, Latvia; 3 SINTEF Industry, Sustainable Energy Technology, NO-7465 Trondheim, Norway; 4 National Institute of Chemical Physics and Biophysics, 12618 Tallinn, Estonia

**Keywords:** biomass, activated carbons, black liquor, wood charcoal, porous structure, supercapacitors

## Abstract

Herein, we present a synthesis route for high-efficiency nitrogen-doped carbon materials using kraft pulping residue, black liquor, and wood charcoal as carbon sources. The synthesized nitrogen-doped carbon materials, based on black liquor and its mixture with wood charcoal, exhibited high specific surface areas (SSAs) of 2481 and 2690 m^2^ g^−1^, respectively, as well as a high volume of mesopores with an average size of 2.9–4.6 nm. The nitrogen content was approximately 3–4 at% in the synthesized nitrogen-doped carbon materials. A specific capacitance of approximately 81–142 F g^−1^ was achieved in a 1 M Na_2_SO_4_ aqueous solution at a current density of 0.2 A g^−1^. In addition, the specific capacitance retention was 99% after 1000 cycles, indicating good electrochemical stability.

## 1. Introduction

Recently, highly efficient energy storage devices have become the focus of significant research efforts [1,2,3,4]. For clean and renewable energy, innovations in supercapacitor (SC) technology are necessary for advanced energy storage systems. A large body of research on SCs has been published [5,6,7,8,9,10,11,12] and this large interest in SCs is driven by their outstanding properties, including high power density, long cycle life, fast charge/discharge rate, eco-friendly nature, flexibility, and lightness. Their broad spectrum of possible applications ranges from portable electronic devices to hybrid electric vehicles [12,13,14,15].

SC performance and successful widespread commercialization is dependent mainly on the electrode material properties which rely heavily on the selection and fabrication of relevant, cheap, effective, and efficient raw materials [12,16,17]. Various novel advanced nanostructured materials, including hybrid derivatives of two or more components coupled with high surface area carbon-based materials or conductive polymers, have been intensively studied [18,19,20,21]. 

Electrode surface area is an important factor to boost the capacitive performance of SCs. Porous and activated carbon, carbon nanotubes, graphene, carbon fibers, and graphitic carbon are the most widely used materials because of their suitable physical and chemical properties [22,23,24]. 

SC performance depends on the surface structure, porosity, and nature of the raw materials as well as the doping of carbon materials with heteroatoms such as N, O, B, S, or P [6,11]. Doping is an alternative method to increase *C*_s_ and creates low-resistance electron transfer channels by reducing ion transfer resistance and diffusion path lengths. Simultaneously, the heteroatom-doped structure provides a large number of active sites for electrochemical reactions, resulting in increased *C*_s_ of the SC material. 

Although most high-quality conventional carbon materials have excellent properties, they are derived from fossil fuels using energy-intensive synthesis techniques, their properties are dependent on the raw material purity, and are time consuming and costly to produce. The ever-growing demand for green energy requires the development of inexpensive and efficient electrode materials for SCs. Biomass-derived carbon is a viable alternative to conventionally produced carbon as it is a renewable source of functional carbon materials for next-generation energy storage systems [25,26,27]. Biomass resources are preferred as carbon precursors because they are renewable, environmentally friendly, abundant, have unique structures, are non-toxic, sustainable, and easy to fabricate. Biomass can be converted into various carbon materials such as porous activated carbon, carbon nanotubes, carbon quantum dots, and heteroatom- or metal-doped carbon via physical or chemical activation methods, including hydrothermal techniques [25,27]. Considerable research has shown that a wide range of biomass sources can be used to produce efficient carbon materials for SCs [28]. In particular, biowaste [29,30], wood tar using crab [31], lignocelluloses [32], wood [33,34,35], and others have been demonstrated as excellent sources of biomass-derived carbon materials for SCs. Recently, wood-derived carbon materials have received the great attention because wood is a biodegradable, renewable material with a naturally hierarchical porous structure and good mechanical performance. Wood-based materials for SCs electrodes, in particular self- or externally/artificially heteroatom-doped electrodes are characterized by enhanced conductivity, surface wettability, and improved capacitance [36]. 

The main goal of the work is to combine two wood processing wastes: solid charcoal (WC) and liquid aromatic lignin (BL), in an alkaline solution as a precursor for the synthesis of micro-, mesoporous carbon material and its use as supercapacitor electrodes. The structure, morphology, and composition of the newly prepared carbon materials were characterized by transmission electron microscopy (TEM), Raman spectroscopy, and X-ray photoelectron spectroscopy (XPS), whereas the electrochemical performances of them were evaluated using 1 M Na_2_SO_4_.

## 2. Materials and Methods

**Figure 1:**
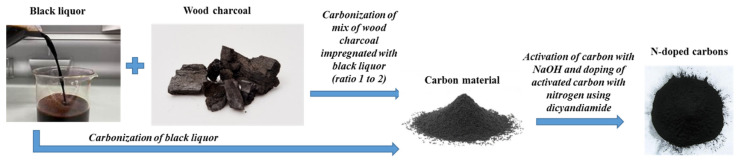
Carbon-based materials fabrication scheme.

Synthesized nitrogen-doped carbon materials using BL from Horizon Pulp & Paper Ltd. Supplier (Kehra, Estonia) and its mixture with WC from Fille Ltd. Supplier (Riga, Latvia) were named as NC-ABL and NC-ABL-WC, respectively. Activation (activator ratio to carbon 3 to 1, activation temperature 800 °C) and modification with nitrogen (dicyandiamide ratio to carbon 20:1, dimethyl formamide as solvent, doping temperature800 °C) are described in detail elsewhere [37]. The carbon-based materials fabrication scheme is shown in Figure 1.

The porous structure of synthesized carbon materials was studied using a Nova 4200e instrument (Quantachrome Instruments, Boynton Beach, FL, USA), as described previously [38]. Specific surface area was determined using BET theory, micropores volume was calculated using Dubinin-Raguskevich theory, and pores size distribution was assessed using Density Functional Theory. 

TEM analysis of the samples was performed using a Tecnai G2 F20 X-TWIN microscope (FEI, Eindhoven, The Netherlands) [39].

The chemical composition of the carbon samples was analyzed by XPS utilizing a Kratos AXIS Supra+ spectrometer (Kratos Analytical, Manchester, UK) as described in more detail elsewhere [40]. 

Raman spectra were recorded using an inVia Raman spectrometer (Renishaw, Wotton-under Edge, UK) equipped with a thermoelectrically cooled (−70 °C) CCD camera and microscope, as described elsewhere [41], except that the laser power at the sample was restricted to 0.2 mW to prevent sample damage. The excitation wavelength was 532 nm. Parameters of the Raman bands were determined by fitting the experimental contour with Gaussian and Lorentzian–Gaussian form components by using GRAMS/A1 8.0 (Thermo Scientific, Waltham, MA, USA) software.

All electrochemical measurements were performed using a three-electrode cell with cyclic voltammetry (CV). A Zennium electrochemical workstation (Zahner-Elektrik GmbH & Co.KG, Kronach—Gundelsdorf, Germany) was used. The prepared NC-ABL and NC-ABL-WC samples were coated on a titanium (Ti) sheet with a geometric surface area of 1 cm^2^ and employed as the working electrode. A Pt sheet was used as the counter electrode and Ag/AgCl/KCl (3 M KCl) as the reference electrode. The carbon material inks were prepared as follows: 5 mg of each sample was dispersed ultrasonically in 0.250 mL of 2% polyvinylidenefluoride (PVDF) in an N-methyl-2-pyrrolydone (NMP) solution for 1 h. Then, the obtained slurry was sprayed onto the Ti electrode and dried in an oven at 80 °C for 2 h. The active material mass loading was 1.1 and 1.5 mg_cat_ cm^−2^ for the NC-ABL and NC-ABL-WC samples, respectively. 

Cyclic voltammograms (CVs) were recorded in a 1 M Na_2_SO_4_ solution at scan rates between 5 and 100 mV s^−1^. All solutions were deaerated with argon (Ar) for 15 min prior to the measurements. A galvanostatic charge/discharge (GCD) test was performed in a two-electrode configuration separated with a glass fiber filter (ROTILABO^®^ Type: CR259) for each of the carbon materials. The electrode material was prepared by mixing 80 wt% of synthesized NC-ABL or NC-ABL-WC carbons, 10 wt% polyvinylidene fluoride (PVDF), and 10 wt% carbon black. Thereafter, N-methylpyrrolidone (NMP) and ethanol were added to the mixture to make a slurry. After stirring for 12 h, the mixture was dropped onto titanium plates and the prepared electrodes were dried at 60 °C. The mass per area of the single electrode in the cell was approximately 1.25 mg cm^−2^. The specific capacitance *Cs* (F g^−1^) values were calculated from the GCD test according to Equation (1) [42]:

*Cs* = 2*I* Δ*t*/m ΔV (1)

where *C*_s_ is the specific capacitance (F g^−1^), *I* is the current in the charge–discharge process (A), m is the active material mass (g), ΔV is the voltage change in the supercapacitor between completely charged and discharged (V), and Δ*t* is the discharged time.

The electrochemical impedance spectroscopy (EIS) spectra were obtained at OCP in the frequency range from 100 kHz to 100 mMHz or 10 mHz, with a perturbation amplitude of 10 mV. The electrochemical impedance data were modeled with equivalent electric circuits (EEC) using Zview software version 3.4.

## 3. Results

This study investigated the synthesis and properties of N-doped porous carbon materials based on BL, WC, and BL composites. BL is produced in the kraft-pulping process by splitting the bonds of wood components, which generally consists of lignin and a small portion of hemicelluloses dissolved in an aqueous solution of sodium hydroxide (NaOH) and sodium sulfide (Na_2_S). In our case, BL refers to an aqueous solution of lignin residues, hemicellulose, and inorganic chemicals used in the kraft-pulping process (11.2% NaOH, 10.9% Na_2_CO_3_, and 21.9% NaHCO_3_).

**Figure 2:**
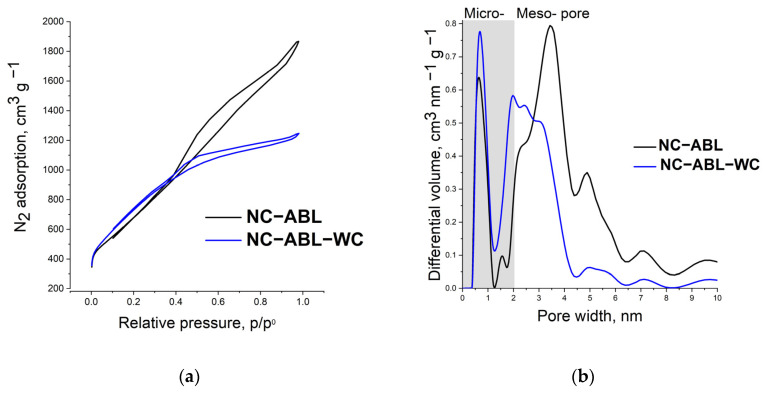
N_2_ adsorption–desorption isotherms (**a**) and pore size distribution (**b**) of the NC-ABL and NC-ABL-WC samples.

An important property of N-doped carbons is the pore size distribution, which determines the surface area and mass transfer of chemicals and ionic groups. The porous structures of the obtained carbon materials were studied using nitrogen sorption at 77 K, and the isotherms are shown in Figure 2a. The pore size distributions calculated according to quenched solid density functional theory (QSDFT) are shown in Figure 2b. The surface areas were calculated according to Brunauer–Emmett–Teller (BET) theory and are provided in Table 1. In Figure 2a, the NC-ABL adsorption–desorption isotherm forms a hysteresis, indicating significant mesopore presence in the porous structure. In the case of NC-ABL-WC, only a small hysteresis is observed, from which it can be concluded that both materials are micro-, mesoporous—in the case of NC-ABL-WC, the volume of micro- and mesopores is almost equal, and for NC-ABL—with the predominance of mesopores The structure and pore size distribution of the samples prepared herein were related to the chemical composition and structure of the raw material.

When liquid BL was used as a raw material, the specific surface area obtained during activation was smaller than when the WC and BL composite was used due to the formation of larger pores, with SSAs of 2481 (NC-ABL) and 2690 m^2^ g^−1^ (NC-ABL-WC). Although the micropore volume, which directly affects the SSA, is similar for both samples, the increased total pore volume input of mesopores and reaches a maximum for the NC-ABL sample at 2.1 m^3^ g^−1^ or 71.6% of the total pore volume. It should be noted that the mesopore volume was higher and average pore size increased (4.7 and 2.9 nm for NC-ABL and NC-ABL-WC, respectively).

**Figure 3:**
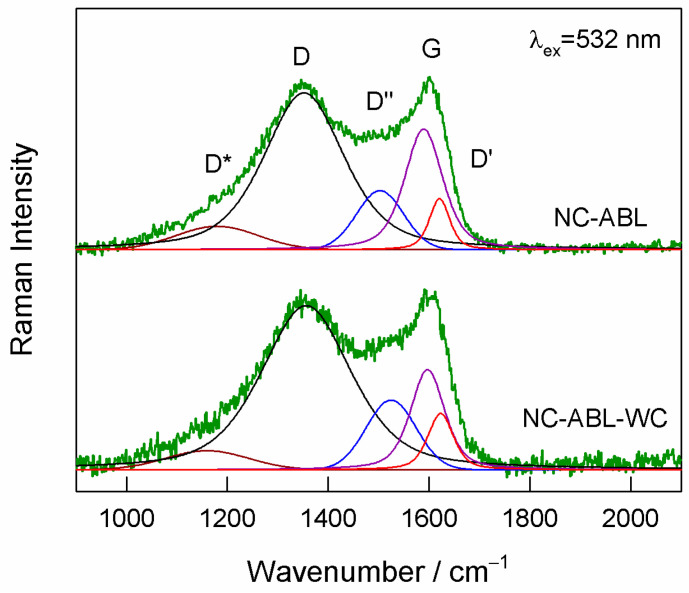
Raman spectra of the NC-ABL and NC-ABL-WC carbon samples using an excitation wavelength of 532 nm (0.2 mW). The fitted Gaussian (D* and D″) and Lorentzian–Gaussian (D, G, and D′) form components are also shown.

Resonance Raman spectroscopy was used to obtain insights into the structure of the newly prepared carbon materials. The two strong bands visible at 1350–1352 and 1601–1602 cm^−1^ belong to the prominent D and G modes of the graphite structure, respectively [43,44] (Figure 3). Both samples exhibited high background in the vicinity of 1100–1200 and 1500–1540 cm^−1^. A more detailed analysis of such complex spectra of carbon material can be performed by fitting the experimental contour with five components [45]. The band denoted as D’ has been ascribed to the disorder induced by crystal-defects, while the broad features denoted as D* and D″ bands were related with a disorder in graphitic lattice and the presence of amorphous carbon materials, respectively [45,46]. Claramunt et al., suggested that relative intensity I(D)/I(D) + I(G) of fitted components provides a measure of the crystallite size (L_α_) on basal planes [45]. The dependence of this ration on the inverse value of crystallite size exhibited relatively good agreement with Cuesta model [47] for disordered carbon-based material [45]. We found that the intensity ratio I(D)/I(D) + I(G) increases from 56 to 62% comparing samples NC-ABL and NC-ABL-WC, respectively. In addition, the relative intensity of D″ band is higher in the case of sample NC-ABL-WC (Figure 3). Thus, spectral analysis revealed that the structure of NC-ABL-WC sample is slightly more disordered compared with NC-ABL.

**Figure 4:**
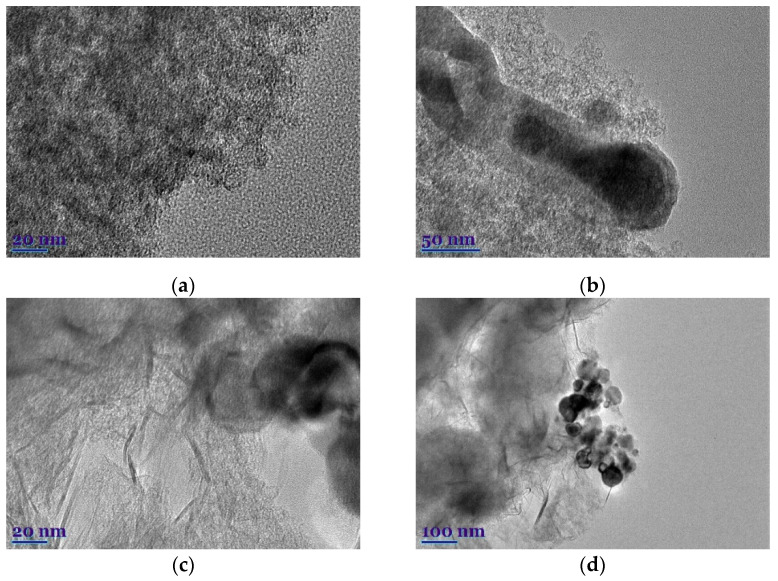
TEM images of the NC-ABL (**a**,**b**) and NC-ABL-WC (**c**,**d**) carbon samples under different magnifications.

TEM images of the NC-ABL and NC-ABL-WC carbon samples under different magnifications are shown in Figure 4. The high-resolution TEM images of both samples showed the existence of micropores and mesopores, indicating an efficient synthesis process.

**Figure 5:**
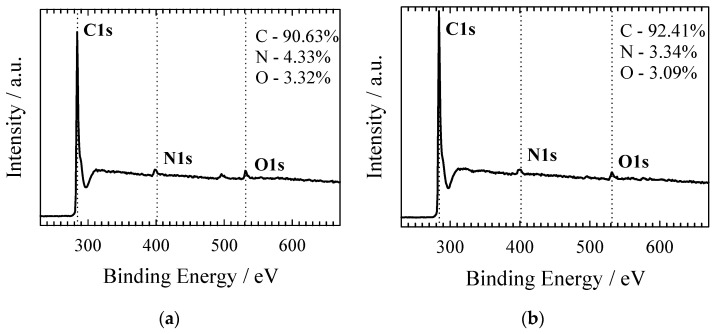
Survey spectra of NC-ABL (**a**) and NC-ABL-WC (**b**) carbons.

The surface elemental compositions and element-binding configurations of the NC-ABL and NC-ABL-WC samples were investigated by XPS. Figure 5 shows representative XPS survey spectra for the NC-ABL (a) and NC-ABL-WC (b) samples, demonstrating the existence of N, C, and O.

**Figure 6:**
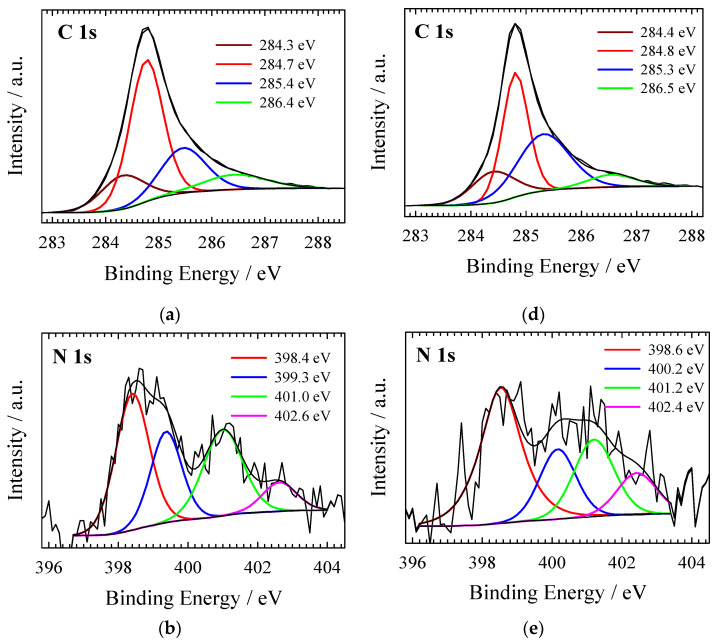

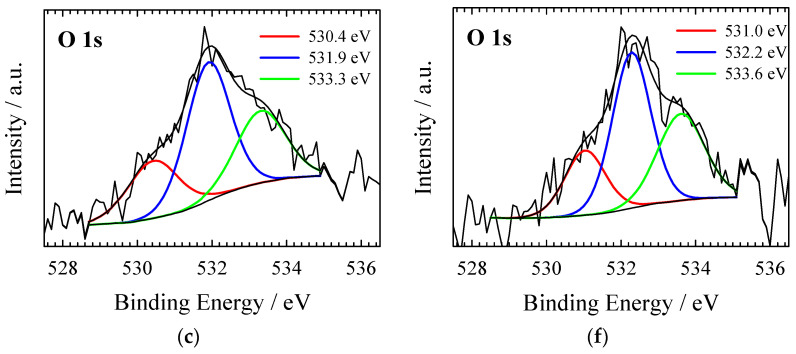
XPS spectra of NC-ABL (**a**–**c**) and NC-ABL-WC (**d**,**e**) carbons of C 1s (**a**,**d**), N 1s (**b**,**e**), and O 1s (**c**,**f**).

The deconvoluted XPS spectra of C 1s, N 1s, and O 1s for NC-ABL and NC-ABL-WC carbon samples are shown in Figure 6.

The high-resolution C 1s XPS spectra of NC-ABL and NC-ABL-WC carbon samples are divided into four peaks at 284.3–284.4 (Csp^2^, 15.88–16.36 at%), 284.7–284.8 (Csp^3^, 51.55–40.00 at%), 285.4–285.3 (N-sp^2^-C, 22.44–36.49 at%), and 286.4–286.5 (N-sp^3^-C, 10.13–7.15 at%) eV [48,49,50]. The N 1s peaks show four peaks identified as pyridinic-N (398.4–398.6 eV, 38.87–36.83 at%), pyrrolic-N (399.3–400.2 eV, 24.03–28.40 at%), graphitic-N (401.0–401.2 eV, 28.78–24.62 at%), and oxidized-N (402.6–402.4 eV, 8.32–10.15 at%). The overall nitrogen content of the NC-ABL and NC-ABL-WC carbon samples was 4.33 and 3.34 at%, respectively, but most was in the pyridinic-N form, which is widely recognized as an ORR-active species [51,52]. The O 1s spectra can be deconvoluted into three peaks at 530.4–531.0, 531.9–532.2, and 533.3–533.6 eV corresponding to C=O, C-OH phenolic hydroxyl/C-O-C ether, and COOH [53,54]. Oxygen functional groups greatly enhance the specific capacitance by introducing Faraday pseudo-capacitance [54].

**Figure 7:**
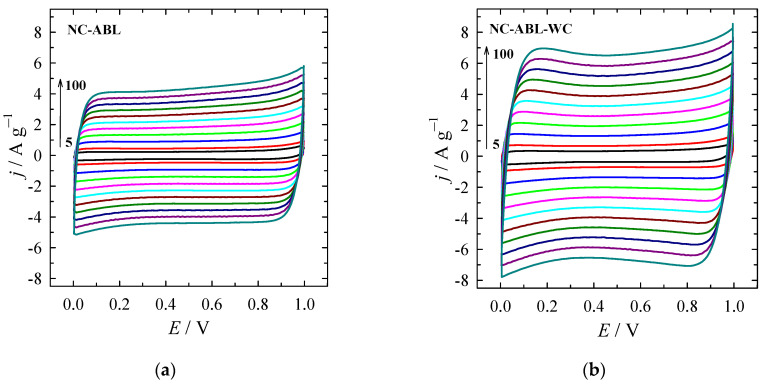

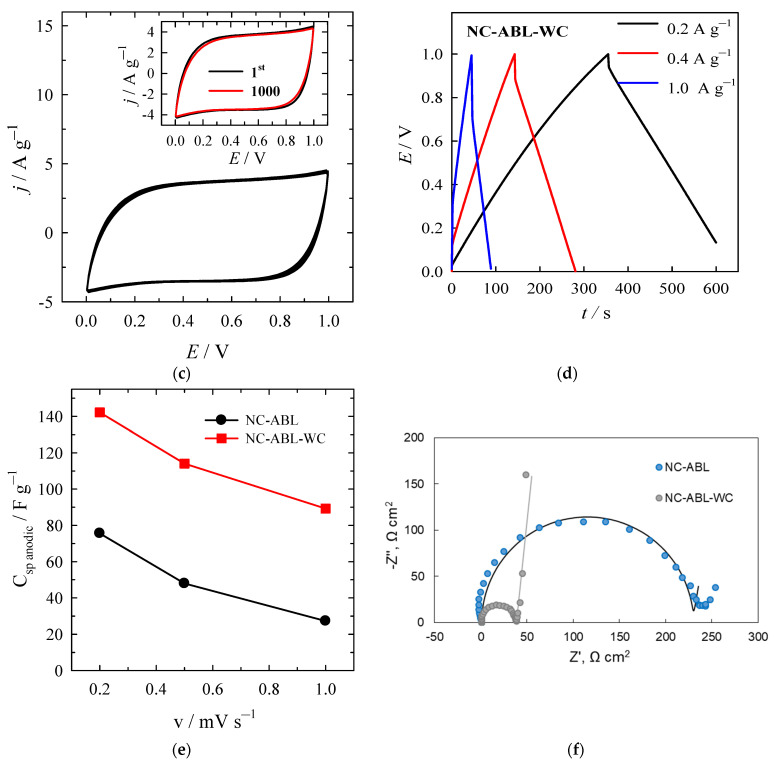
CV curves of the NC-ABL (**a**), NC-ABL-WC (**b**) carbon electrodes recorded in 1 M Na_2_SO_4_ solution at scan rates of 5–100 mV s^−1^. CVs for NC-ABL at 100 mV s^−1^ (**c**) (the inset represents CVs after 1st and 1000 cycles). GCD profiles (**d**) and specific capacitances (**e**) at a different current density of NC-ABL-WC and NC-ABL measured in a two-electrode system. (**f**) Electrochemical impedance spectra of both carbon materials under the frequency range from 100 kHz to 100 mMHz or 10 mHz, with a perturbation amplitude of 10 mV.

Figure 7 presents the electrochemical performance of the NC-ABL and NC-ABL-WC samples using CV at scan rates from 5 to 100 mV s^−1^. Both samples exhibit a rectangular shape, indicative that the specific capacitance is mainly induced by the electrical double layer capacitance (EDLC) and partly by the Faraday pseudo-capacitance (PS) (Figure 7a,b). The long-term stability of the NC-ABL sample was also evaluated by recording CVs at 100 mV s^−1^ for up to 1000 cycles (Figure 7c). The inset represents CVs after 1st and 1000 cycles. The specific capacitance retention was 99% after 1000 cycles, indicating good long-term electrochemical stability of the electrode.

The GCD performance of the NC-ABL and NC-ABL-WC samples was evaluated in a symmetrical two-electrode cell. Figure 7d shows the GCD curves for NC-ABL-WC at different current densities. At the current density of 0.2 A g^−1^, the NC-ABL-WC device shows a higher specific capacitance of 142.23 F g^−1^ as compared with the device with NC-ABL sample (80.93 F g^−1^) (Figure 7e). The performance of electrode materials can be influenced by the shape of pores and their size distribution as well as by particular types of pores (micro- and mesopores) in their total volume [55,56]. Micropores up to 2 nm in size, accessible to electrolyte ions, provide high capacitive characteristics of the material, whereas mesopores with sizes of 2–50 nm can play the role of transport channels during the processes of charge and discharge, and thus, their presence leads to a decrease in the resistance of the SC, which is mainly due to the transfer of ions in the porous structure of the electrodes [57,58,59]. Large mesopores, as well as macropores, can serve as a buffer reservoir, due to which the ion diffusion distance can be reduced. The ratio of micropores to mesopores is also an important factor in allowing enhanced capacitance or conductivity [60]. Although both NC-ABL and NC-ABL-WC materials are micro-, mesoporous and showed the same micropore volume, the total pore volume is significantly smaller in the case of NC-ABL (28.4%). The volume of micro- and mesopores is almost equal in the case of NC-ABL-WC, whereas mesopores predominance in the structure is determined for NC-ABL. The higher electrochemical performance of NC-ABL-WC may be attributed to the porous structure, larger accessible surface area, and the volume of micro- and mesopores and advisable proportion of micropore volume to total volume of 56.5%. Additionally, the high activity of nitrogen-doped activated carbon materials may be attributed to the presence of different types of O and N functional groups (such as C=O, C-OH phenolic hydroxyl/C-O-C ether, COOH, pyridinic-N, pyrrolic-N, graphitic-N and oxidized-N), which can directly generate pseudo-capacitance via redox reactions, improve the hydrophilicity and wettability, and conductivity of the activated carbon [53,60]. 

The impedance spectra shown in Figure 7f represent the capacitive behavior of the samples NC-ABL and NC-ABL-AWC. The high-frequency signal deviates from theoretical predictions, possibly due to the developed porous surface of the samples. The spectra were fitted with a typical Randles circuit, but also with an additional CPE element to represent low-frequency capacitance. This element was added for fitting quality but not analyzed. For NC-ABL the impedance magnitude is larger and equivalent circuit fitting data (shown in Table 2) reveal that the cause is mainly its larger R_ct_ value (228.4 Ω cm^2^). In comparison, the R_ct_ of NC-ABL-AWC is 37.7 Ω cm^2,^ and consequently, the impedance is lower. The difference in capacitances is not significant, but for NC-ABL-AWC C_dl_ is larger by ~10 mF cm^2^ in comparison to NC-ABL. In addition, a strong low-frequency capacitive response is seen for NC-ABL-AWC, which can be attributed to the accumulation SO_4_^2-^ on the material’s surface.

**Figure 8:**
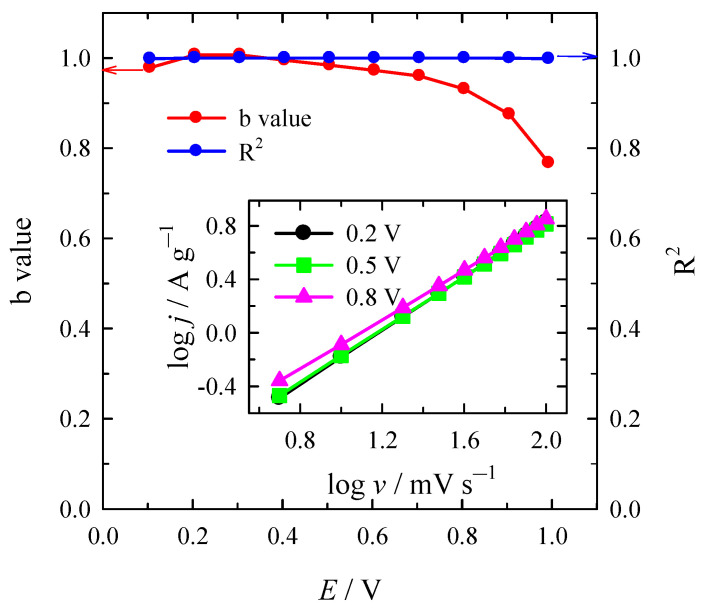
The b value under anode scan and its corresponding fitting variance R^2^ for the NC-ABL-WC.

As was mentioned above, the capacitance of SC can be divided into the double layer capacitance caused by the adsorption–desorption process of the electrolyte ions and the Faraday capacitance induced by the redox reaction on the surface of the material [54,56]. The charge storage mechanism of SC can be evaluated using the following formula [56,61]:

*I* = av^b^ (2)

where the measured current *I* (A g^−1^) at a fixed potential obeys a power law relationship with the scan rate v, both a and b are adjustable parameters. Then b = 0.5, the charge storage mechanism is considered to follow the diffusion control process, and then b = 1, the surface control process is suggested [56]. Parameter b can be determined from the slope of the dependence of log(*j)* vs. log(*v*). Figure 8 presents the obtained b values under anodic scan and its corresponding fitting variance R^2^ of the NC-ABL-WC. The determined b values varies in the range of 0.76 to 1 with the change in potential and the corresponded fitting variance R^2^ is close to 1. This indicates that charge storage mechanism for NC-ABL-WC material is mainly dependent on surface control process [56]. 

Additionally, the CV analysis was performed on Duun’s method [61], allowing evaluating the contribution of the surface control capacitance and the diffusion control capacitance to the total capacitance (Equation (3)):

*j* = *k*_1_*v* + *k*_2_*v*^0.5^ (3)

where, *k*_1_*v* and *k*_2_*v*^0.5^ represent the surface capacitive and diffusion-controlled current contributions, respectively. Dividing the equation by *v*^0.5^ on both sides gives the following Equation (4):

*jv*^−0.5^ = *k*_1_*v*^0.5^ + *k*_2_ (4)

**Figure 9:**
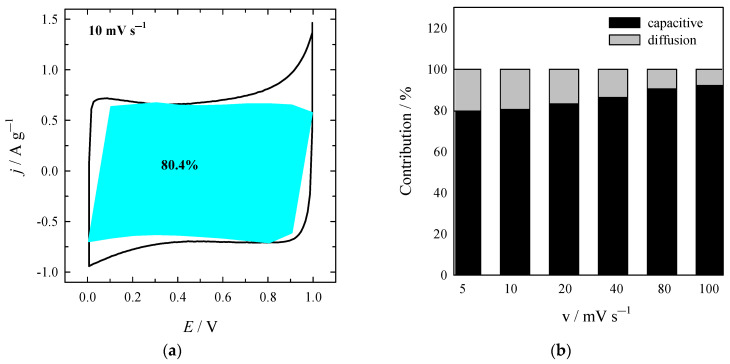
(**a**) The contribution of surface control capacitance at 10 mV s^-1^ for the NC-ABL-WC and (**b**) the columnar contribution map of surface control capacitance at different scan rates.

At a fixed potential, the *jv*^−0.5^ scaled linearly with the square root of scan rates (*v*^0.5^), and gave the slope of *k*_1_ and a y-intercept of *k*_2_. The *k*_1_*v* represents the surface capacitive-controlled current contribution. At other fixed potentials, a series of *k*_1_ and *k*_2_ were obtained. Figure 9a shows the percentage of surface control capacitance contribution to total capacitance at a scan rate of 10 mV s^−1^. The contribution of surface control capacitance reaches 79.6% at 5 mV s^−1^ and 92.0 % at 100 mV s^−1^, indicating a diminution of the contribution of diffusion control capacitance to charge storage capability with the increasing scan rate (Figure 9b). 

A comparison of the supercapacitive behavior of various carbon-based electrode materials reported in the literature and the present work is presented in Table 3, exhibiting the suitable specific capacitance of the newly prepared electrode materials. The capacitance values are close to the capacitance reported for carbon-based materials.

## 4. Conclusions

Herein, we present a simple synthesis route for high-efficiency nitrogen-doped carbon materials using kraft pulping residue, black liquor, and wood charcoal as carbon sources. The synthesized nitrogen-doped carbon materials from black liquor and wood char exhibited high SSAs of 2481 and 2690 m^2^ g^−1^, respectively, and contained a large volume of pores with an average size of 2.9−4.6 nm. The nitrogen content was approximately 3–4 at% in the synthesized carbon materials doped with nitrogen. A specific capacitance of approximately 81−142 F g^−1^ was achieved in a 1 M Na_2_SO_4_ aqueous solution at a current density of 0.2 A g^-1^. In addition, the specific capacitance retention was 99% after 1000 cycles, indicating good electrochemical stability. The obtained results demonstrate that the N-doped activated carbon materials obtained from BL (NC-ABL) and its mixture with WC (NC-ABL-WC) are promising electrode materials for SC applications.

## Figures and Tables

**Table 1:** The porous structure of NC-ABL and NC-ABL-WC carbon samples.

Material	SSA (BET), m^2^ g^−1^	Total Pore Volume (Vt), cm^3^ g^−1^	Micropore Volume, cm^3^ g^−1^	Mesopore Volume, cm^3^ g^−1^	Mesopores from Vt, %	Average Pore Width, (nm)
NC-ABL	2480	2.9	0.8	2.1	71.6	4.7
NC-ABL-WC	2690	1.9	0.8	1.1	56.5	2.9

**Table 2:** Parameters of EIS for NC-ABL-WC and NC-ABL carbon materials.

Material	R_s_, Ω cm^2^	C_dl_, mF cm^2^	R_ct_, Ω cm^2^
NC-ABL-WC	0.83	32.3	37.7
NC-ABL	0.88	26.2	228.4

**Table 3:** Comparison of specific capacitance of various carbon-based materials.

Material	Specific Surface Area, m^2^ g^−1^	Electrolyte	Specific Capacitance, F g^−1^	Current Density, A g^−1^	Ref.
NC-ABL	2481	1 M Na_2_SO_4_	142.23	0.2	This work
NC-ABL-WC	2690	1 M Na_2_SO_4_	80.93	0.2	This work
N-APC-800	623	0.2 M K_2_SO_4_	231	0.1	[55]
Mp-NCF-900	60.8	1 M Na_2_SO_4_	316	0.5	[62]
			168	5.0	
AC	1198	1 M Na_2_SO_4_	93	1.0	[63]
AC	2250	1 M Na_2_SO_4_	135	0.2	[64]
Seaweed carbons LN600	746	0.5 M Na_2_SO_4_	125	0.2	[65]

## Data Availability

Not applicable.
